# Supplementary material for: Spatial Structuring of Soil Fungal Diversity Associated with Ziziphus lotus (Rhamnaceae) in Arid Agricultural Soils
Source: Microorganisms. 2025 Oct 30;13(11):2489. doi: 10.3390/microorganisms13112489 (PMC12654320; doi:10.3390/microorganisms13112489)
Supplement: Supplementary file 1 [file microorganisms-13-02489-s001.zip › microorganisms-3871044-supplementary.pdf]

Supplementary Information

Table S1. Physicochemical characteristics of different *Ziziphus lotus* clusters in barley-planted and non-barley-planted fields across varying sampling distances. EC: electrical conductivity; OM: organic matter; CEC: cation exchange capacity. Cluster codes begin with 'C' for barley-planted fields. followed by the cluster number (1–5) and the sampling distance (0. 3. or 6 m). Similarly, 'T' denotes clusters from unplanted fields. followed by the cluster number (1–2) and the sampling distance.

Cluster	Clay %	Silt %	Sand %	pH	EC ms/cm	$CaCO_3$ %	OM %	P_2O_5 mg/kg	K_2O mg/kg	Na_2O mg/kg	MgO mg/kg	CaO mg/kg	NO_3^- mg/kg	NH_4^+ mg/kg	Total N %	Cu mg/kg	Fe mg/kg	Mn mg/kg	Zn mg/kg	CEC meq/100g
C1-0	24	48	28	8.05	0.63	0.4	2.8	125	1006	189	626	5308	80.4	6.18	0.18	0.69	2.06	11.12	0.76	12
C1-3	34	44	22	8.37	0.19	0.3	2.2	124	851	231	632	5230	57.29	3.12	0.17	0.93	2.41	11.12	0.98	15.5
C1-6	32	46	22	8.69	0.13	0.3	1.66	34	466	238	567	5223	7.86	0.12	0.13	0.72	1.92	6.34	0.37	14.2
C2-0	26	48	26	8.17	0.4	0.5	2.15	67	470	253	609	6177	61.25	3.52	0.17	0.74	1.78	9.95	0.59	12.1
C2-3	28	50	22	8.7	0.13	0.3	1.71	31	327	244	566	4563	7.17	0.16	0.13	0.7	1.82	5.92	0.44	12.6
C2-6	30	50	20	8.66	0.11	0.3	1.72	27	317	196	578	4857	3.29	0.02	0.13	0.8	2.12	7.67	0.37	13.4
C3-0	24	44	32	8.14	0.27	0.6	2.81	70	654	294	629	6070	73.49	3.07	0.18	0.68	1.77	13.51	0.74	11.8
C3-3	28	52	20	8.57	0.16	0.5	2.18	37	410	224	567	5628	12.51	0.2	0.17	0.65	1.76	6.31	0.38	12.9
C3-6	30	50	20	8.48	0.15	0.6	1.77	32	406	200	558	5308	9.52	0.05	0.14	0.8	2.11	8.79	0.39	13.2
C4-0	26	48	26	8.18	0.31	1.1	3.2	98	700	155	652	6360	63.94	4.1	0.19	0.66	1.72	13.92	0.75	12.5
C4-3	26	52	22	8.38	0.22	0.8	1.78	44	448	283	667	6687	14.91	0.83	0.14	0.76	2.1	8.79	0.51	11.4
C4-6	30	50	20	8.58	0.18	0.7	1.68	40	401	265	565	5817	12.34	1.11	0.13	0.75	2.19	7.45	0.48	13
C5-0	30	42	28	8.17	0.27	0.5	2.91	90	570	230	647	4901	60.08	1.16	0.18	0.6	1.55	9.92	0.76	14.4
C5-3	26	50	24	8.62	0.12	4	1.93	35	375	207	544	4759	9.49	0.14	0.15	0.69	1.71	6.6	0.39	11.9
C5-6	26	52	22	8.66	0.12	0.5	1.68	35	359	216	539	5661	6.75	0.06	0.13	0.68	1.65	5.92	0.35	11.6
T1-0	24	42	34	8.22	23	0.5	2.53	136	791	122	507	4797	58.21	1.31	0.17	0.87	2.11	18.61	1.06	11.6
T1-3	22	50	28	8.36	0.22	0.5	1.71	43	352	200	439	3911	30.31	0.11	0.13	0.66	1.73	5.81	0.44	10

T1-6	22	50	28	8.35	0.17	0.6	1.53	37	339	197	493	4597	12.89	4.49	0.12	0.67	1.93	5.87	0.43	9.8
T2-0	22	50	28	8.06	0.29	0.6	2.53	72	619	185	575	6556	57.08	3.04	0.17	0.68	1.76	20.11	0.59	10.8
T2-3	26	52	22	8.33	0.18	0.5	1.48	39	411	190	506	6352	40.67	0.55	0.11	0.68	1.71	5.61	0.37	11.4
T2-6	30	50	20	8.6	0.15	0.5	1.46	30	354	180	500	5238	24.54	0.06	0.11	0.76	1.96	4.69	0.42	12.9

Table S2. Results of multivariate ANOVA showing the influence of soil physicochemical properties on microbial community structure. Significance levels (*p*-values) are indicated. with asterisks denoting statistical significance (*$p \leq 0.05$. **$p \leq 0.01$. ***$p \leq 0.001$).

Variable	Diversity	Sum_Sq	Mean_Sq	F_value	*p*_value	Significance
Clay	Shannon	0.049	0.049	0.608	0.439	
Silt		0.100	0.100	1.235	0.271	
pH		0.060	0.060	0.741	0.393	
EC		0.106	0.106	1.305	0.258	
$CaCO_3$		0.382	0.382	4.705	0.034	*
Organic matter		0.302	0.302	3.723	0.058	
P_2O_5		0.017	0.017	0.213	0.646	
K_2O		0.186	0.186	2.292	0.135	
Na_2O		1.027	1.027	12.646	0.001	***
MgO		0.080	0.080	0.989	0.324	
CaO		0.030	0.030	0.370	0.545	
NO_3^-		0.672	0.672	8.279	0.006	**
NH_4^+		0.049	0.049	0.598	0.442	
Total N		0.004	0.004	0.052	0.821	
Cu		0.004	0.004	0.049	0.825	
Fe		0.096	0.096	1.187	0.280	
Mn		0.023	0.023	0.279	0.599	
Zn		0.014	0.014	0.169	0.682	
CEC		0.193	0.193	2.371	0.129	
Residuals		4.791	0.081	NA	NA	
Clay	Simpson	0.002	0.002	2.077	0.155	
Silt		0.001	0.001	0.884	0.351	
pH		0.001	0.001	1.527	0.221	
EC		0.000	0.000	0.186	0.668	
$CaCO_3$		0.004	0.004	4.657	0.035	*
Organic matter		0.003	0.003	3.136	0.082	
P_2O_5		0.001	0.001	0.630	0.431	
K_2O		0.000	0.000	0.016	0.900	
Na_2O		0.010	0.010	11.265	0.001	***
MgO		0.001	0.001	1.549	0.218	
CaO		0.000	0.000	0.223	0.639	
NO_3^-		0.004	0.004	4.760	0.033	*
NH_4^+		0.000	0.000	0.075	0.786	
Total N		0.000	0.000	0.014	0.905	
Cu		0.000	0.000	0.043	0.837	
Fe		0.000	0.000	0.385	0.538	
Mn		0.001	0.001	1.000	0.322	
Zn		0.000	0.000	0.090	0.765	
CEC		0.003	0.003	3.782	0.057	
Residuals		0.051	0.001	NA	NA	

Table S3. Results of univariate ANOVA demonstrating the influence of soil physicochemical properties on microbial community structure. Significance levels (*p*-values) are indicated. with asterisks denoting statistically significant differences (*$p \leq 0.05$. **$p \leq 0.01$. ***$p \leq 0.001$).

Variable	Diversity	Test	Statistic	*p*_value	Significance
Clay	Shannon	Regression	0.467	0.496	
Silt		Regression	1.202	0.276	
Sand		Regression	0.080	0.778	
pH		Regression	0.471	0.495	
EC		Regression	0.364	0.548	
$CaCO_3$		Regression	2.399	0.125	
Organic matter		Regression	5.286	0.024	*
P_2O_5		Regression	1.638	0.204	
K_2O		Regression	1.137	0.290	
Na_2O		Regression	6.290	0.014	*
MgO		Regression	14.839	0.000	***
CaO		Regression	0.934	0.337	
NO_3^-		Regression	2.537	0.115	
NH_4^+		Regression	0.154	0.696	
Total N		Regression	7.576	0.007	**
Cu		Regression	0.004	0.952	
Fe		Regression	0.320	0.573	
Mn		Regression	0.402	0.528	
Zn		Regression	2.766	0.100	
CEC		Regression	2.226	0.140	
Clay	Simpson	Regression	1.716	0.194	
Silt		Regression	1.201	0.277	
Sand		Regression	0.047	0.830	
pH		Regression	0.300	0.586	
EC		Regression	0.182	0.671	
$CaCO_3$		Regression	1.798	0.184	
Organic matter		Regression	4.700	0.033	*
P_2O_5		Regression	2.510	0.117	
K_2O		Regression	3.063	0.084	
Na_2O		Regression	5.690	0.020	*
MgO		Regression	18.892	0.000	
CaO		Regression	4.167	0.045	*
NO_3^-		Regression	2.540	0.115	
NH_4^+		Regression	1.007	0.319	
Total N		Regression	7.010	0.010	**
Cu		Regression	0.359	0.551	
Fe		Regression	0.087	0.769	
Mn		Regression	1.039	0.311	
Zn		Regression	3.403	0.069	
CEC		Regression	4.349	0.040	*

Table S4. Rarefaction analysis of the samples showing the original sequence reads and corresponding diversity indices.

Samples	Samples.1	Distance_m	Original_Reads	Rarefied_Reads	Observed	Chao1	Shannon	Simpson
14	Planted	0m	5195	1103	63	69.875	3.255858	0.929452
26	Planted	0m	9658	1103	90	102.3529	3.545433	0.952493
38	Planted	0m	5256	1103	59	68.42857	3.030266	0.9088
50	Planted	0m	3767	1103	56	65	3.224615	0.934566
62	Non-Planted	0m	2637	1103	35	35.75	2.581298	0.893359
74	Non-Planted	0m	1567	1103	31	33	2.383022	0.862821
3	Planted	0m	1971	1103	48	50	3.134711	0.934657
15	Planted	0m	4486	1103	58	71	3.101661	0.919618
27	Planted	0m	5752	1103	57	61	3.186448	0.935413
13	Planted	0m	2823	1103	50	52.8	2.986517	0.916531
39	Planted	0m	5767	1103	70	83.6	3.30715	0.942419
51	Planted	0m	5912	1103	72	80.66667	3.241423	0.905599
63	Non-Planted	0m	4952	1103	52	56	2.900468	0.911302
75	Non-Planted	0m	3042	1103	56	64.66667	2.980695	0.924904
4	Planted	0m	1756	1103	34	36	2.865844	0.917029
16	Planted	0m	3540	1103	57	61.66667	3.293261	0.943963
28	Planted	0m	5239	1103	73	85	3.414119	0.944039
40	Planted	0m	3923	1103	61	69.66667	3.065039	0.918691
52	Planted	0m	3954	1103	59	68.16667	3.221985	0.930904
25	Planted	0m	2569	1103	30	44	2.516933	0.888956
64	Non-Planted	0m	3134	1103	56	58.625	3.135039	0.92507
76	Non-Planted	0m	3178	1103	53	59	2.997111	0.914815
5	Planted	3m	1448	1103	33	38	2.911754	0.922502
17	Planted	3m	4077	1103	52	55.5	2.935258	0.902975
29	Planted	3m	7286	1103	71	75.875	3.242956	0.931645
41	Planted	3m	4457	1103	60	71	3.206728	0.933743
53	Planted	3m	3712	1103	54	61.85714	3.123278	0.930095
65	Non-Planted	3m	5146	1103	61	66.5	2.978986	0.90146
77	Non-Planted	3m	2094	1103	39	44.25	2.734845	0.890608
37	Planted	0m	2359	1103	35	45.5	2.569206	0.876148

6	Planted	3m	6406	1103	85	95.11111	3.729341	0.961428
18	Planted	3m	4081	1103	54	61.2	3.198457	0.939901
30	Planted	3m	3205	1103	39	84	2.669663	0.892065
42	Planted	3m	3349	1103	49	61	3.0087	0.917634
54	Planted	3m	3947	1103	70	83	3.300714	0.93407
66	Non-Planted	3m	4739	1103	67	80	3.078285	0.898826
78	Non-Planted	3m	2336	1103	56	60.66667	3.145436	0.925946
7	Planted	3m	5065	1103	38	40.5	2.882759	0.921657
49	Planted	0m	1880	1103	47	49.5	2.88367	0.908956
19	Planted	3m	7565	1103	80	87.5	3.45748	0.94084
31	Planted	3m	4001	1103	57	68	3.030841	0.910618
43	Planted	3m	5921	1103	63	67.5	3.326875	0.940769
55	Planted	3m	5203	1103	56	58.625	3.309687	0.947304
67	Non-Planted	3m	3873	1103	31	33	2.025981	0.74505
79	Non-Planted	3m	5111	1103	51	58.5	2.98362	0.925489
8	Planted	3m	2196	1103	42	44.625	2.935551	0.926523
20	Planted	3m	5413	1103	54	56	3.070886	0.919341
32	Planted	3m	5105	1103	52	53.75	3.064738	0.930458
61	Non-Planted	0m	1756	1103	40	47	2.791641	0.881471
44	Planted	3m	3706	1103	56	63.5	3.081724	0.924162
56	Planted	3m	2492	1103	52	61.16667	2.935685	0.903372
68	Non-Planted	3m	2440	1103	54	58	3.097942	0.927151
80	Non-Planted	3m	3142	1103	40	45.25	2.626995	0.886313
9	Planted	6m	3839	1103	48	52.66667	2.980704	0.912472
21	Planted	6m	3947	1103	52	56.2	3.183293	0.939809
33	Planted	6m	3217	1103	31	32.2	2.335264	0.845718
57	Planted	6m	4048	1103	44	46.14286	2.970723	0.928338
73	Non-Planted	0m	3019	1103	46	48	2.93254	0.915747
69	Non-Planted	6m	1721	1103	32	35.33333	2.4404	0.848148
81	Non-Planted	6m	4613	1103	59	63.58333	3.037702	0.919681
10	Planted	6m	2845	1103	46	68	2.857777	0.912992
22	Planted	6m	4351	1103	77	82.2	3.429142	0.933896
34	Planted	6m	2037	1103	36	36.33333	2.627522	0.868055

46	Planted	6m	2263	1103	36	43	2.809303	0.915592
70	Non-Planted	6m	1477	1103	37	40	2.899883	0.918022
82	Non-Planted	6m	2080	1103	32	34	2.380396	0.858115
11	Planted	6m	1191	1103	20	20.5	2.392315	0.887289
23	Planted	6m	3362	1103	61	65	3.092294	0.907759
35	Planted	6m	2941	1103	45	48.75	3.001525	0.92494
47	Planted	6m	3942	1103	38	45.5	2.803421	0.908448
59	Planted	6m	2918	1103	39	42.33333	2.593623	0.875285
71	Non-Planted	6m	1103	1103	30	30.33333	2.611819	0.891703
83	Non-Planted	6m	1304	1103	33	40	2.697867	0.894825
12	Planted	6m	1611	1103	35	49	2.458288	0.845927
24	Planted	6m	3715	1103	46	53.5	2.923774	0.911678
36	Planted	6m	1238	1103	24	24.33333	2.300257	0.853844
48	Planted	6m	2445	1103	47	54	3.02388	0.926084
60	Planted	6m	1563	1103	34	41	2.70664	0.899921
72	Non-Planted	6m	2273	1103	35	50	2.323819	0.833176

Table S5: Top 20 fungal ASVs shared across distance (0 m, 3 m, 6 m) identified by random forest.

0m	3m	6m	Mean Decrease Accuracy	Mean Decrease Gini	ASV	Kingdom	Phylum	Class	Order	Family	Genus
10.6	4.5	5.8	10.6	1.3	ASV_96	Fungi	Ascomycota	Dothideomycetes	Pleosporales	Dothidotthiaceae	NA
8.1	6.7	3.3	8.5	0.9	ASV_8	Fungi	Ascomycota	Dothideomycetes	Pleosporales	Dothidotthiaceae	Pleiochaeta
8.4	1.7	4.8	8.4	1.0	ASV_93	Fungi	Ascomycota	Dothideomycetes	Dothideales	Saccotheciaceae	Aureobasidium
4.4	4.9	7.1	8.3	0.9	ASV_62	Fungi	Ascomycota	Sordariomycetes	Sordariales	Chaetomiaceae	NA
9.5	5.1	-3.1	7.5	0.9	ASV_106	Fungi	Ascomycota	Dothideomycetes	Pleosporales	Pleosporaceae	NA
9.4	-2.5	4.9	7.4	1.1	ASV_122	Fungi	Ascomycota	Dothideomycetes	Pleosporales	Pleosporaceae	Pleosporaceae_gen_Incertae_sedis
6.1	4.0	0.8	6.2	0.6	ASV_24	Fungi	Ascomycota	Dothideomycetes	Pleosporales	Didymellaceae	NA
4.6	3.1	3.3	6.2	0.7	ASV_60	Fungi	Ascomycota	Sordariomycetes	Hypocreales	Nectriaceae	Fusarium
5.3	3.2	4.0	6.0	0.5	ASV_30	Fungi	Ascomycota	Pezizomycetes	Pezizales	Ascobolaceae	Ascobolus

5.0	4.5	0.3	5.5	0.4	ASV_11	Fungi	Ascomycota	Dothideomycetes	Pleosporales	Didymellaceae	Xenodidymella
4.5	4.6	3.0	5.4	0.3	ASV_696	Fungi	Ascomycota	Dothideomycetes	Pleosporales	Didymellaceae	NA
3.9	5.0	-0.3	5.2	0.4	ASV_98	Fungi	Ascomycota	Dothideomycetes	Pleosporales	Didymellaceae	NA
4.0	4.8	1.7	5.2	0.4	ASV_933	Fungi	Ascomycota	Dothideomycetes	Pleosporales	Pleosporaceae	Alternaria
6.9	-0.5	2.0	5.0	0.7	ASV_51	Fungi	Ascomycota	Dothideomycetes	Pleosporales	Didymellaceae	NA
4.6	3.8	2.6	5.0	0.3	ASV_50	Fungi	Ascomycota	Dothideomycetes	Venturiales	Sympoventuriaceae	Ochroconis
6.5	0.9	1.2	5.0	0.7	ASV_5	Fungi	Ascomycota	Dothideomycetes	Pleosporales	Didymellaceae	NA
4.7	0.6	4.3	5.0	0.5	ASV_254	Fungi	Ascomycota	Sordariomycetes	Sordariales	Chaetomiaceae	NA
4.6	4.5	0.0	4.9	0.5	ASV_12	Fungi	Ascomycota	Dothideomycetes	Cladosporiales	Cladosporiaceae	Cladosporium
6.1	-1.2	3.2	4.7	0.8	ASV_6	Fungi	Ascomycota	Dothideomycetes	Pleosporales	Pleosporaceae	Alternaria
4.2	2.6	2.3	4.5	0.3	ASV_34	Fungi	Ascomycota	Dothideomycetes	Dothideales	Saccotheciaceae	Aureobasidium

Figure S1.

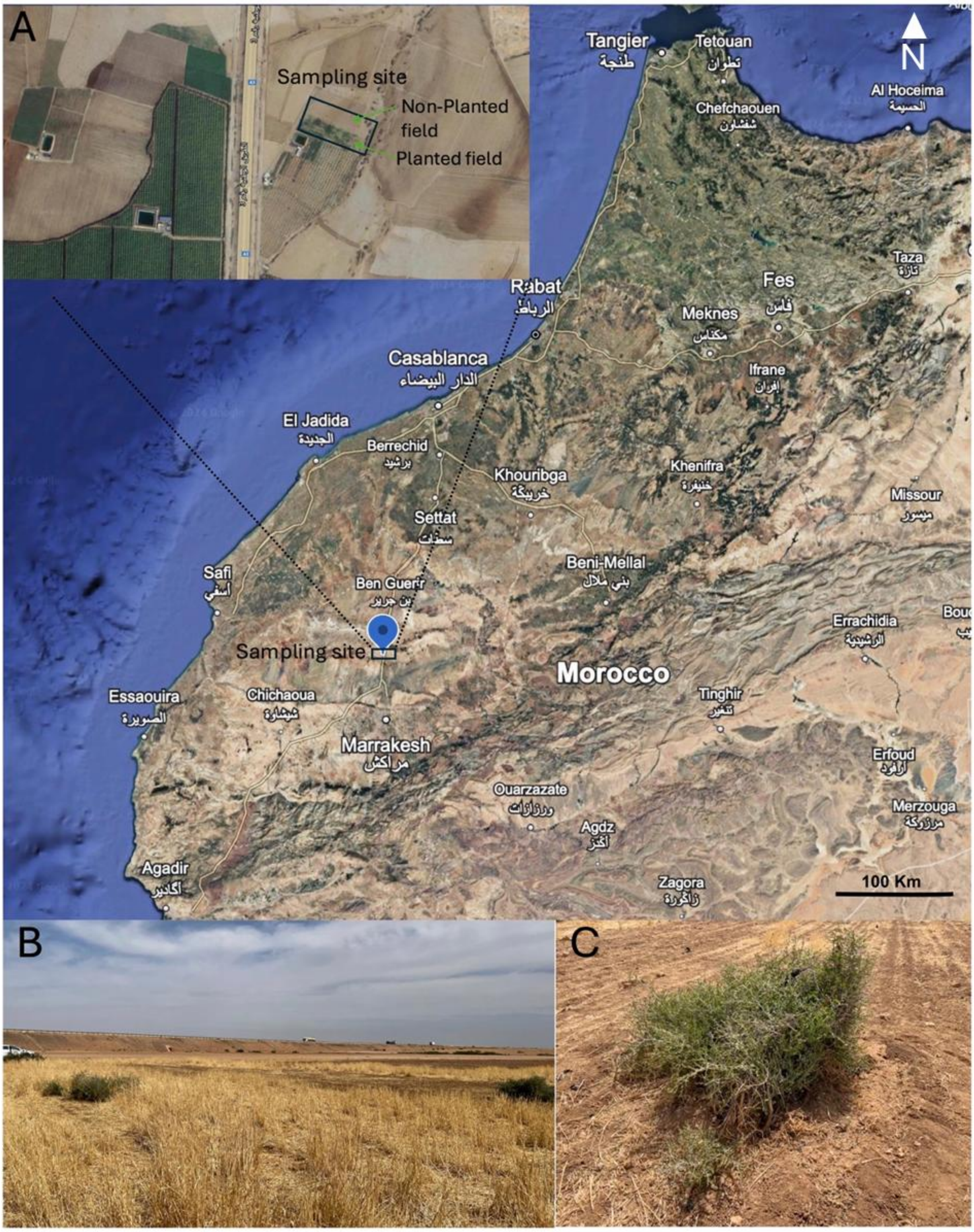

Figure S1. (A) Location of the sampling site in Rhamna Province. between Ben Guerir and Marrakech. Morocco retrieved from Google Maps. along the A3 highway. (B) Image of a barley-planted field with patches of wild jujube shrubs. (C) Close-up view of a wild jujube shrub patch.

Figure S2.

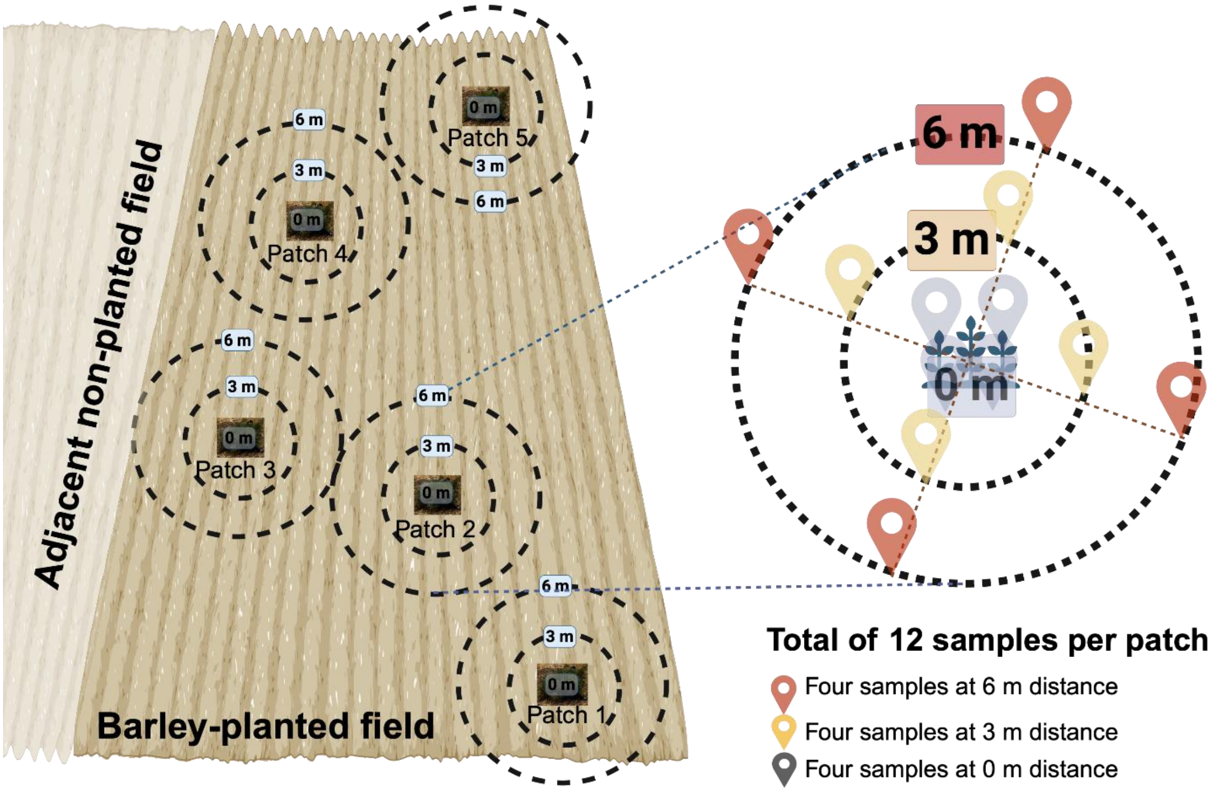

Figure S2. Experimental sampling in barley-planted and non-planted fields. The diagram illustrates the sampling design. with soil samples collected at three distances (0 m. 3 m. and 6 m) from *Ziziphus lotus* patches. Each sampling cluster consists of 12 samples.

Figure S3.

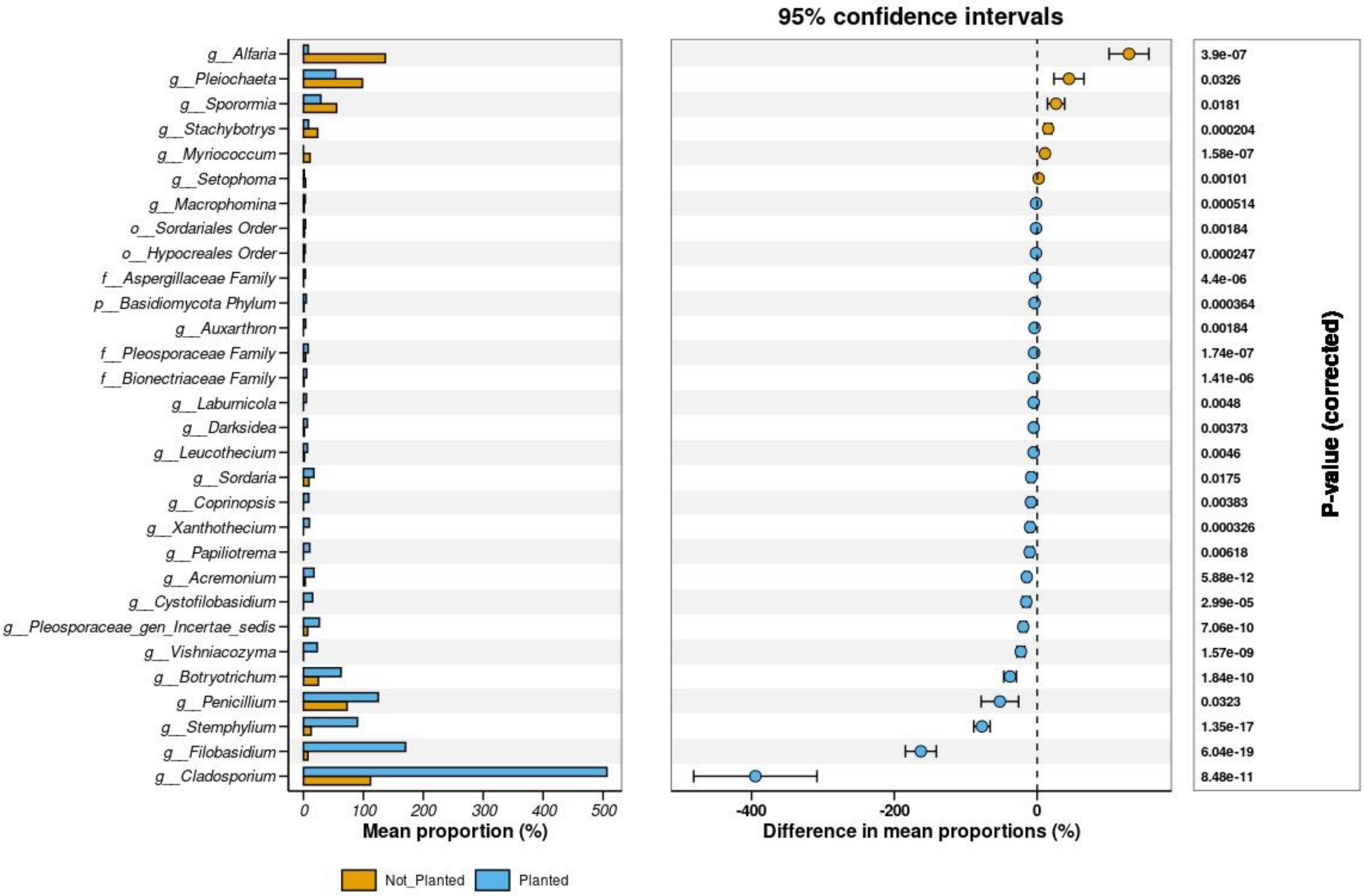

Figure S3. Differential abundance analysis of fungal taxa between planted and non-planted fields.

Figure S4.

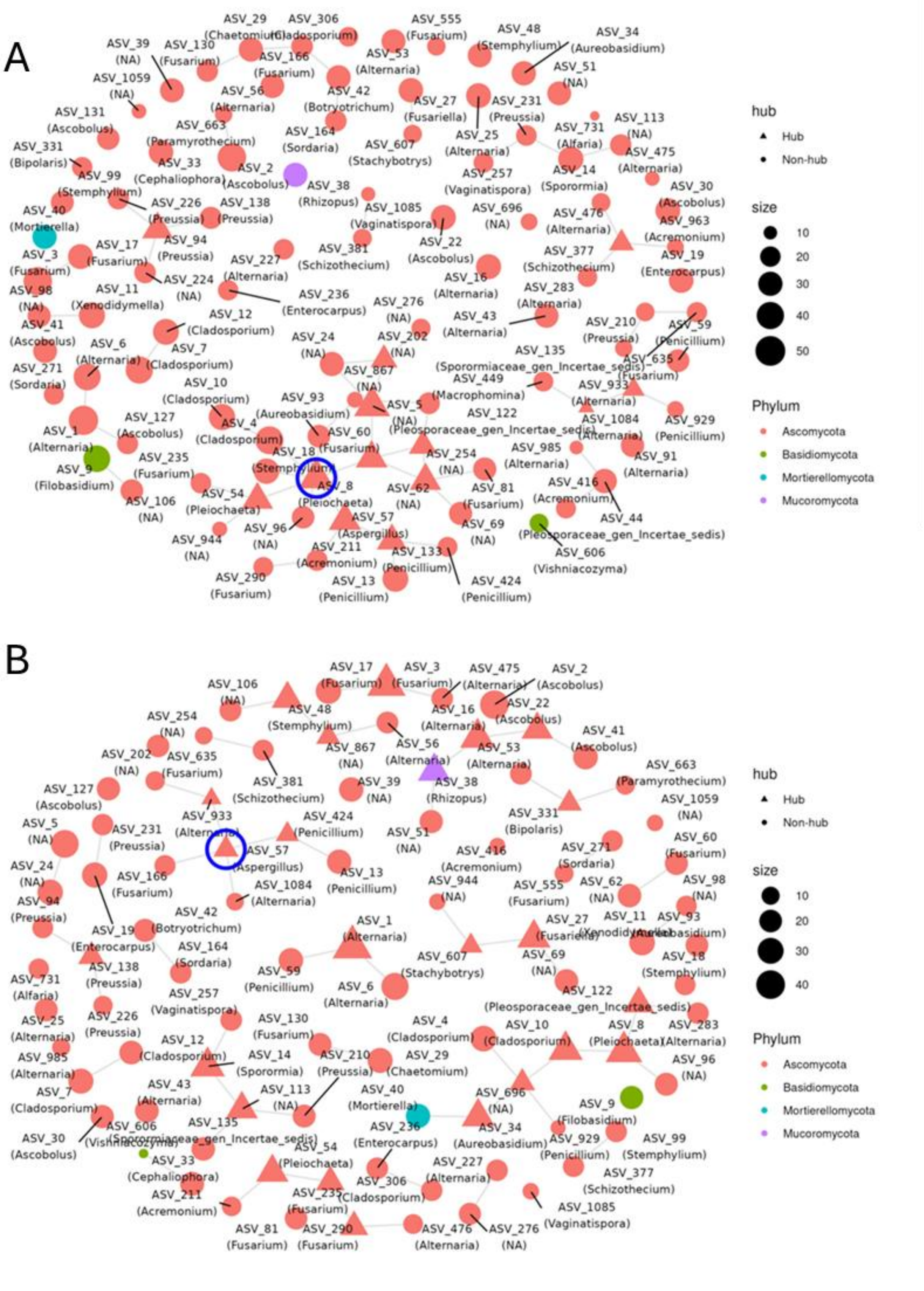

Figure S4. Cooccurrence analysis of planted and non-planted sampling sites. the triangles are the hub taxa over top ASV in 10% of the samples. the key taxa are encircled in blue. node size indicate the abundance of taxa.
